# The Mechanism of Anticorrosion Performance and Mechanical Property Differences between Seawater Sea-Sand and Freshwater River-Sand Ultra-High-Performance Polymer Cement Mortar (UHPC)

**DOI:** 10.3390/polym14153105

**Published:** 2022-07-30

**Authors:** Tianyu Li, Xin Sun, Fangying Shi, Zheng Zhu, Dezhi Wang, Huiwen Tian, Xiaoyan Liu, Xunhuan Lian, Tengfei Bao, Baorong Hou

**Affiliations:** 1 College of Water Conservancy and Hydropower Engineering, Hohai University, Nanjing 210098, China; 20220916@hhu.edu.cn (T.L.); 170802020008@hhu.edu.cn (Z.Z.); 2 Key Laboratory of Marine Environmental Corrosion and Bio-Fouling, Institute of Oceanology, Chinese Academy of Sciences, Qingdao 266071, China; tianhuiwen@qdio.ac.cn (H.T.); baoronghou@163.com (B.H.); 3 College of the Environment, Hohai University, Nanjing 210098, China; 2014010202@hhu.edu.cn; 4 School of Civil and Hydraulic Engineering, Ningxia University, Yinchuan 750021, China; wangdzh@nxu.edu.cn; 5 College of Mechanics and Materials, Hohai University, Nanjing 210098, China; 211308030002@hhu.edu.cn

**Keywords:** sea sand, polymer cement mortar, UHPC, anticorrosion analysis, material characterization, X-CT

## Abstract

There are abundant sea-sand resources on the earth. Traditional sea-sand concrete faced various problems relating to insufficient anticorrosion ability. In this paper, artificial seawater, sea sand, industrial waste, steel fiber, and polycarboxylate superplasticizer were used to prepare ultra-high-performance polymer cement mortar (SSUHPC). At the same time, freshwater river-sand ultra-high-performance polymer cement mortar (FRUHPC) with the same mixing ratio was prepared for comparative study. The compressive strength of SSUHPC reached 162.1 MPa, while the that of FRUHPC reached 173.3 MPa, which was slightly higher. Meanwhile, SSUHPC showed excellent anticorrosion characteristics in terms of carbonization, frost resistance and chloride resistance, and especially for sulfate resistance. The composition of SSUHPC was separated into three parts: mortar, pore and steel fiber, and the performance difference mechanisms of SSUHPC and FRUHPC were investigated by X-ray computed tomography (X-CT), mercury intrusion porosimetry (MIP), scanning electron microscopy (SEM), and X-ray diffraction (XRD). The hydration degree of mortar in SSUHPC was higher, with higher content of CSH and CH, and its better optimized gel pore characteristics gave SSUHPC better corrosion resistance. The mechanical properties of SSUHPC were slightly poor due to the uneven dispersion of steel fibers and air pores, with an- air pore porosity of 1.52% (above 200 μm) that was twice that of FRUHPC (0.6%). In this paper, the mechanics and anticorrosion performance of ultra-high-performance polymer cement mortar prepared with seawater sea sand were comprehensively evaluated, and the mechanism of performance difference between SSUHPC and FRUHPC was revealed, conducive to the targeted improvement of sea sand concrete.

## 1. Introduction

At present, river sand is the main source of sand for construction, but the supply of river sand is limited by resources and environmental impact, and cannot fully met the demands of the construction industry. China’s coastal areas are rich in sea-sand resources, and the total amount of sea sand in China’s offshore regions is about 67.96 × 10^10^~68.49 × 10^10^ m^3^ [1]. The mining of sand for construction has gradually shifted to the marine environment. The orderly development, research, and utilization of abundant sea-sand resources as construction aggregates has become an inevitable development trend. 

Ultra-high performance concrete (UHPC) was developed by Hodia, Lafarge and Bouygues, and was registered as a patent by Ductal [2]. Compared with conventional concrete, UHPC has many advantages: the compressive strength of UHPC is higher than 150 MPa, which is about three times greater than conventional concrete. UHPC has excellent toughness and fracture energy. Compared with high-performance concrete and some metal equivalents, the toughness of UHPC was found to increase by more than 300 times [3,4,5,6]. In addition, UHPC has good durability [7,8,9] and low permeability [10,11], which can substantially improve its service life and reduce the maintenance costs of concrete structures [7,12,13,14,15]. Due to its high performance standards, UHPC has been widely used in the construction and rehabilitation of infrastructure. The most popular application of UHPC in North America has been as grouting material for bridge connections and bridge rehabilitation [16,17,18]. To date, domestic and international scholars have conducted extensive research on UHPC. Norhasri et al. [19] added nano-kaolinite as an additive to UHPC and found that nano-kaolinite UHPC blends had lower workability effects due to their clay properties and ultrafine size compared with normal UHPC and metakaolinite UHPC, but nano-kaolinite UHPC exhibited similar compressive strengths to normal UHPC and metakaolin UHPC in the early stages, which gradually increased in the later stages. Tanarslan [20] studied the performance of reinforced concrete (RC) beams reinforced with prefabricated ultra-high-performance fiber-reinforced concrete (UHPFRC) laminates, and the UHPFRC-reinforced specimens showed a minimum increase of 32% and a maximum increase of 208% in load-carrying capacity. Therefore, the use of UHPFRC laminates can enhance the performance of reinforced concrete beams, and was an effective technique for improving load-bearing capacity.

Sea-sand concrete is a kind of concrete mixed with sea sand as fine aggregate, which has been used in coastal areas on a large scale in recent years [21,22,23]. The use of sea sand can solve the problem of river-sand shortages to a certain extent, and has the advantage of being inexpensive. However, sea sand contains a large quantity of chloride salts. Chloride ions have been identified as a key factor in the corrosion of reinforcing steel used in reinforced concrete, and an important cause of reduced durability in concrete. The chloride ions (Cl−) in sea sand may threaten concrete properties if it is not treated prior to use [24,25,26,27]. Many studies at home and abroad have shown that seawater sea sand can be used in concrete, and high mechanical properties can be maintained by adding external admixtures (fly ash, slag, steel fiber, etc.) [28,29,30,31,32,33,34,35,36,37]. Li et al. [38] used seawater sea sand to prepare reactive powder concrete, and found that the carbonation of reactive powder concrete prepared from seawater sea sand was mainly the due to the reaction of CO_2_ with Ca(OH)_2_, C-S-H gel, C_3_S, and C_2_S. The generated CaCO_3_ with amorphous hydrated SiO_2_ covered the surface of the original concrete, to increase the carbonation resistance of the concrete. The combination of sea-sand concrete and UHPC prepared into sea-sand high-performance concrete also has practical engineering significance. Li et al. [39] and Zhang et al. [40] studied the mechanical properties, early workability, and high temperature resistance of seawater sea-sand high-performance concrete. They found that compared with freshwater river-sand steel fibers, the mechanical properties of the seawater sea sand concrete were not inferior, and the concrete prepared using seawater sea sand had better fire resistance and blast resistance properties. Li et al. [41] prepared a high-performance concrete (SHPC) using sea sand and simulated seawater, which had good resistance to freezing, chlorination, carbonation, and sulfate. To investigate the service performance of SHPC, they placed it in seawater for one year. Their test results showed that SHPC demonstrated advantages such as good performance and durability. The construction period of SHPC was short, making it suitable for promotion and application.

X-ray computed tomography (X-CT) is a non-destructive testing technique that uses X-rays as the energy source to obtain images of the internal structure of an object through computer reconstruction. It can be used in the construction field to study the hydration of cement, characterization of pore structure, mortar interface transition zone, geometric distribution of fibers in concrete, sulfate erosion, corrosion of reinforcement, carbonation, freeze–thaw damage, cracking, etc. It is suitable for observing the internal microstructure of concrete [42,43,44,45,46,47,48]. Shi et al. [49] obtained a three-dimensional model of the pore structure based on the X-CT technique, visualizing the effect of desert sand on the pore structure of fiber-reinforced mortar. Wang et al. [47] used an X-ray CT system to study the spatial distribution of steel fibers and bubbles in cylindrical UHPC specimens. Jiang et al. [50] discovered the deformation and cracking patterns of cement mortar coverings during corrosion by using a 16 mm resolution X-CT scanning technique. The three-dimensional model established using X-ray CT and image analysis techniques allowed visualization and quantitative analysis of the distribution of steel fibers and air bubbles in concrete samples. Zhou [51] et al. investigated the deterioration performance and safety of sea-sand concrete by analyzing the CT characteristics of different parts and regions of sea-sand concrete. Li et al. [52] investigated the sulfate corrosion performance of UHPC with seawater sea-sand and freshwater river-sand, and the degree of internal damage after six months was confirmed with the help of X-ray CT technology.

Traditional sea-sand concrete is faced with various durability problems and cannot achieve the breakthrough improvement of anti-erosion performance. The specific performance of UHPC prepared with seawater and sea sand remains unknown, and so too do the performance difference mechanism between that and traditional UHPC. UHPC has excellent mechanical and durability characteristics. This study designed and prepared two kinds of UHPC, using seawater sea sand and freshwater river sand, respectively. The mechanical properties, fatigue resistance and durability of SSUHPC and FRUHPC were compared and studied. The differences between SSUHPC and FRUHPC were systematically studied by X-CT, SEM, XRD, and MIP techniques. Finally, the mechanism of the performance difference between UHPCs prepared with seawater sea sand and with freshwater river-sand was proposed.

## 2. Materials and Methods

### 2.1. Raw Materials

The materials used in this study include ordinary Portland cement P·O 42.5 from Nanjing Conch Cement Co., Ltd. (Nanjing, China), silica fume from Gongyi Yuan Heng water purification material Factory, fly ash from Gongyi Yuan Heng water purification material factory, polycarboxylic acid series superplasticizer from Shandong Yuncheng Brilliant New Building Materials Technology Co., Ltd., copper-plated straight steel fiber from Ganzhou Daye Metallic Fibres Co., Ltd., (Ganzhou, China), river sand and sea sand. The basic physical properties and chemical compositions of the cement, silica fume, and fly ash were determined by X-ray fluorescence (XRF) (Table 1, Table 2, Table 3 and Table 4). The water consumption for standard consistency and setting time of the cement were tested according to Chinese standard GB/T1346-2019; the fineness of the cement was tested according to Chinese standard GB/T 1345-2005; the density of the cement was tested according to Chinese standard GB/T 208-2014; the compressive strength and flexural strength of the cement were tested according to Chinese standard GB/T 17671-2021. The basic physical properties of the fly ash were tested according to Chinese standard GB/T1596-2017. The basic physical properties of the silica fume were tested according to Chinese standard GB/T 27690-2011. The steel fibers were 13 mm long, with a diameter of 0.2 mm, a length–diameter ratio of 65, and tensile strength greater than or equal to 2850 MPa. The solid content of the superplasticizer was 40%, with a water-reduction rate of 35~40%. Sea sand was purchased from Zhangzhou, Fujian Province, with a fineness modulus of 2.3–2.6, a mud content of less than 1.0%, and a chloride ion concentration of 0.08%. The mud content of the river sand was 1.5%, with a fineness modulus of 2.2–2.5. An artificial seawater solution with a chloride concentration of 3.5% was prepared using analytically pure sodium chloride, composed of NaCl 24.53 g/L, MgCl_2_ 5.20 g/L, Na_2_SO_4_ 4.09 g/L, CaCl_2_ 1.16 g/L, KCl 0.695 g/L, NaHCO_3_ 0.201 g/L, KBr 0.101 g/L, H_3_BO_3_ 0.027 g/L, SrCl_2_ 0.025 g/L, and NaF 0.003 g/L, according to ASTM D1141-98. Either artificial seawater or fresh water was used as mixing water. The physical and chemical characteristics of raw materials and species of raw materials were the same as those published by our team [52].

### 2.2. Mixtures, Specimens Preparation and Exposure Conditions

To investigate the effect of the use of seawater sea sand and freshwater river sand on the performance difference of ultra-high-performance polymer cement mortar (UHPC), two types of UHPC were prepared as shown in Table 5. Sand and steel fiber were added after mixing the cement, fly ash, and silica fume, and the mixture was stirred well. Water and superplasticizer were added during the process of stirring at room temperature. The concrete slurry was loaded into the model and moved on to the follow-up treatments after 24 h. The follow-up treatments included a curing condition of 85 °C hot water for 48 h. SSUHPC refers to the UHPC including sea water and sea sand with 85 °C hot water curing 48 h. FRUHPC refers to the UHPC including fresh water and river sand with 85 °C hot water curing for 48 h. Sample sizes under different test conditions are specified in Section 2.3.

### 2.3. Test Methods

#### 2.3.1. Compressive Strength and Vickers Hardness

Compressive strength of specimens was determined on subsamples with a size of Φ50 × 50 mm^2^, cut from the specimens after curing with a size of 100 mm × 100 mm × 100 mm. The compressive tests were carried out on an Electric Universal Testing Machine with a maximum capacity of 100 kN. The final compressive strength data was the average of three parallel subsamples, and the error margin was 5%. Thin subsamples with a size of Φ50 × 10 mm^2^ were cut from different depths of the specimens for Vickers hardness measurements. In the cutting process, the samples were cut to a slightly larger size and then polished with abrasive paper to a standard thickness of 10 mm. In addition, to avoid the influence of humidity on concrete hardness, the polished samples were placed in an oven at 45 °C for 24 h. Vickers hardness was measured every 0.5 mm from the exposed surface to the interior using a micro-hardness tester (No. HDX-1000TC). The max load was 30 kN, the load holding time 15 s, and the objective lens multiple was 40. To obtain more representative results, eight measurements of Vickers hardness uniformly distributed on a circle at the same depth were recorded for each sample. Then, the maximum and minimum values were removed, and the average of the remaining values taken as the representative value. The final Vickers hardness data was the average of three parallel samples.

#### 2.3.2. Fatigue Test

The fatigue test was carried out by three-point composite beam loading, in line with JTG/T 3364-02-2019. The stress control mode was adopted, and the specimen size was 400 mm × 100 mm × 100 mm.

**Figure 1:**
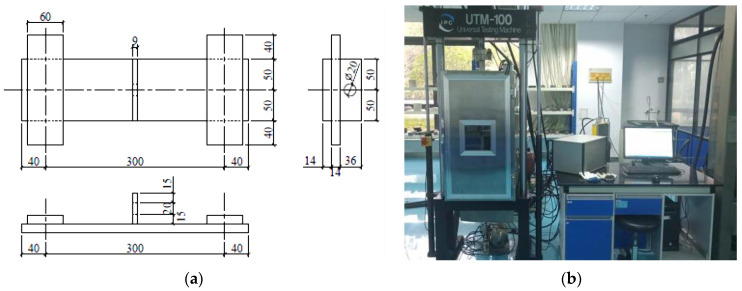
Three-point loading fatigue test device. (**a**) Schematic diagram of three-point loading test; (**b**) UTM-100 fatigue testing machine.

UTM-100 fatigue testing machine was used for the test as shown in Figure 1b, and the system automatically collected the displacement, stress and strain. Firstly, the mid-span load P = 5 kN was set for fatigue testing of the two groups of UHPC. The fatigue loading times were set at 1 million cycles. After the loading times were completed, the appearance changes of SSUHPC and FRUHPC were observed and recorded. If there was no damage, the mid-span fatigue test load was increased to P = 10 kN, and the same loading times were set. The specific scheme is shown in Table 6.

#### 2.3.3. Durability

SSUHPC and FRUHPC species with the same size of 100 mm × 100 mm × 400 mm were placed in fresh water, 3.5 wt% and 7.0 wt% NaCl solutions from (−18 ± 2) °C to (5 ± 2) °C for freeze–thaw cycles, each cycle of 2~4 h. The relative dynamic elastic modulus (Pi) of concrete was measured with the measuring instrument (DT-W18) before and after 1000 freeze–thaw cycles. The relative dynamic modulus was calculated by the following equation according to the Chinese standard SL/T 352-2020:

(1) $$P_{i}=\frac{f_{\mathrm{ni}}^{2}}{f_{0i}^{2}}\times 100$$

(2) $$P=\frac{1}{3}\sum_{i=1}^{3}P_{i}$$

where P was the relative dynamic elastic modulus after 1000 freeze–thaw cycles (%); $P_{i}$ was the relative dynamic elastic modulus of the No. *i* concrete after 1000 freeze–thaw cycles (%); $f_{\mathrm{ni}}^{2}$ was the transverse fundamental frequency of the No. *i* concrete after 1000 freezing-thawing cycles (Hz); $f_{0i}^{2}$ was the transverse fundamental frequency of the No. *i* concrete before 1000 freeze–thaw cycles (Hz).

The concrete was immersed in 5 wt% Na_2_SO_4_ + 7 wt% MgSO_4_ compound solution at 25~30 °C for 18 h, and then dried at (80 ± 5) °C for 6 h. The whole process was one cycle, and the whole test covered a total of 300 cycles. The morphologies of the two groups of UHPC were recorded before and after the sulfate erosion test, and the Vickers hardness was measured from the surface of the concrete to its center of volume. The max load was 30 kN, the load holding time 15 s, and the objective lens multiple was 40.

The electric flux method test was based on the chloride ion migration ability of a saturated concrete specimen, proportional to its conductivity. By applying 60 V voltage on both sides of concrete, the coulomb electric quantity of concrete was recorded for 6 h to evaluate the chloride ion permeability of concrete. Rapid electromigration testing was based on the initial current measured at a voltage of 30 V and a reasonable time for electrification at a reasonable voltage. At the end of the experiment, the specimen was split in half and 0.1 mol/L silver nitrate solution was sprayed on the fresh fracture surface to measure the penetration depth of chloride ions.

#### 2.3.4. Microscopic Characterization Analysis

Pore structures (below 200 μm) of SSUHPC and FRUHPC were analyzed by mercury intrusion porosimetry (MIP). Corresponding interior areas of SSUHPC and FRUHPC were selected for SEM tests. All the selected samples for SEM tests were dried at 45 °C for 24 h using an oven and then coated with gold before testing. A Hitachi S-3400N scanning electron microscope was used. SEM images were photographed at an accelerating voltage of 15 kV. XRD tests were conducted on a Bruker D8-Advance model X-ray diffraction analyser. The Cu-K radiation with a wave length of 1.54 Å was conducted at a voltage of 40 kV under a current of 35 mA. The scanning interval was 2θ = 5–85° with a scanning speed of 2°/min and a step size of 0.02014°.

##### 2.3.5. μX-CT

In this study, the Siemens Somatom Sensation 40 CT machine was adopted to obtain the composition spatial distribution of ingredients and meso-structure information of SSUHPC and FRUHPC species at a size of Φ100 × 100 mm^2^. This X-ray CT system is based on cone-beam scanning technology, which consists of a 240 kV/320 W microfocus X-ray source and a radiation detector with a nominal resolution of less than 2 μm. This microfocused X-ray source has a resolution of 1 μm and a minimum distance of 4.5 mm between the focus and the sample. In the experiment, 190 kV lamp voltage and 0.45 mA current value were used. When the CT system was ready, the cylindrical sample was secured to a table on a low-density poly-cylindrical base. In order to receive the X-ray radiation beam evenly in the acquisition system, each specimen was moved up and down automatically during the 1 h scan.

### 2.4. Modeling and Analysis of SSUHPC/FRUHPC Based on Avizo Software

**Figure 2:**
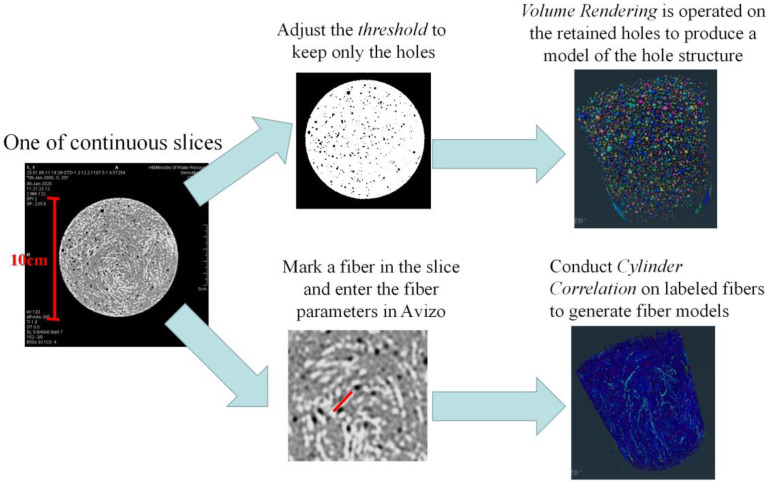
Schematic diagram of the pore and fiber structure reconstruction modeling.

In order to analyze the three-dimensional pore and fiber structure of UHPC made up of seawater sea sand and freshwater river sand, 3D models of the pore and fiber structures of each samples were developed based on continuous slices obtained by μX-CT, and this process was completed by Avizo software. Firstly, the threshold was adjusted and selected, and the pores or fiber were separated from the solid structure to obtain the 3D reconstruction models of the pore and fiber structure of each sample. The pore and fiber structure statistics were obtained through the numerical analysis function included in the software, through which the pore and fiber structure of the sample could be analyzed quantitatively. Figure 2 shows the schematic diagram of the pore and fiber structure reconstruction modeling.

## 3. Results and Discussion

### 3.1. Mechanical Properties and Durability

In order to comprehensively understand the performance of ultra-high-performance polymer cement mortar prepared with seawater and sea sand in this study, the mechanical properties and corrosion resistance of SSUHPC and FRUHPC were compared and studied. The specific research results are as follows.

#### 3.1.1. Mechanical Properties

**Figure 3:**
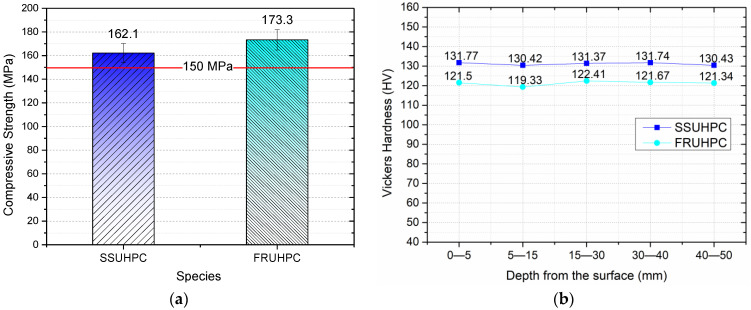
Comparison of compressive strength and Vickers hardness between SSUHPC and FRUHPC: (**a**) Compressive strength; (**b**) Vickers hardness.

After 85 °C hot water curing, the compressive strength of SSUHPC and FRUHPC reached 162.1 ± 8.1 MPa and 173.3 ± 8.6 MPa, respectively, as shown in Figure 3a. The compressive strength of UHPC prepared with seawater sea sand was 93.5% that of FRUHPC, which was slightly reduced. In this study, the compressive strengths both kinds of UHPC were found to be higher than 150 MPa [53].

The mechanical properties of SSUHPC and FRUHPC were further compared by Vickers hardness testing, as shown in Figure 3b. The results showed that there was little difference in the internal Vickers hardness of the UHPC materials, which indicated that the internal structures of the UHPC materials prepared in this study tended to be uniform. However, the Vickers hardness of UHPC materials prepared with sea water and sea sand was higher than that of UHPC materials prepared with fresh water and river sand, which indicated that the hydration degree of mortar was more mature when using seawater and sea sand to prepare UHPC, and the strength of the corresponding hardened cement mortar was higher.

**Figure 4:**
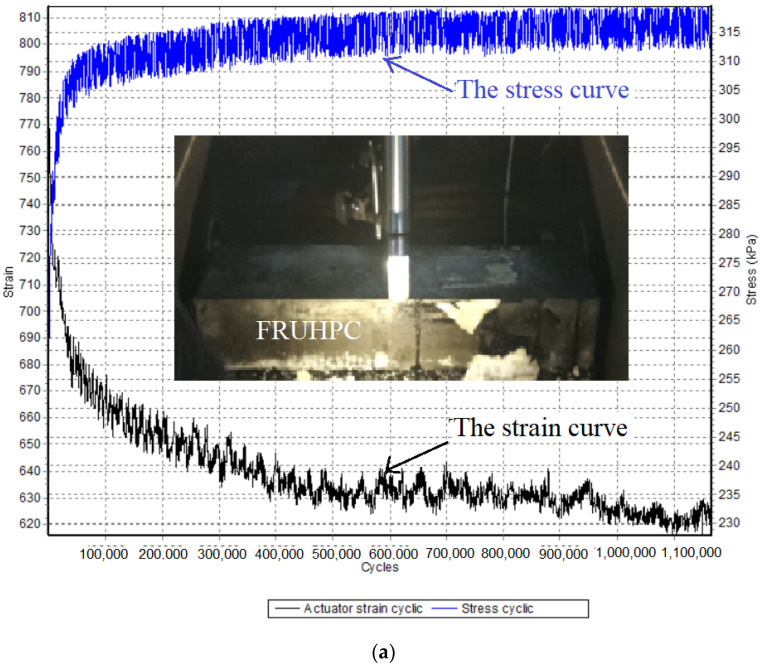

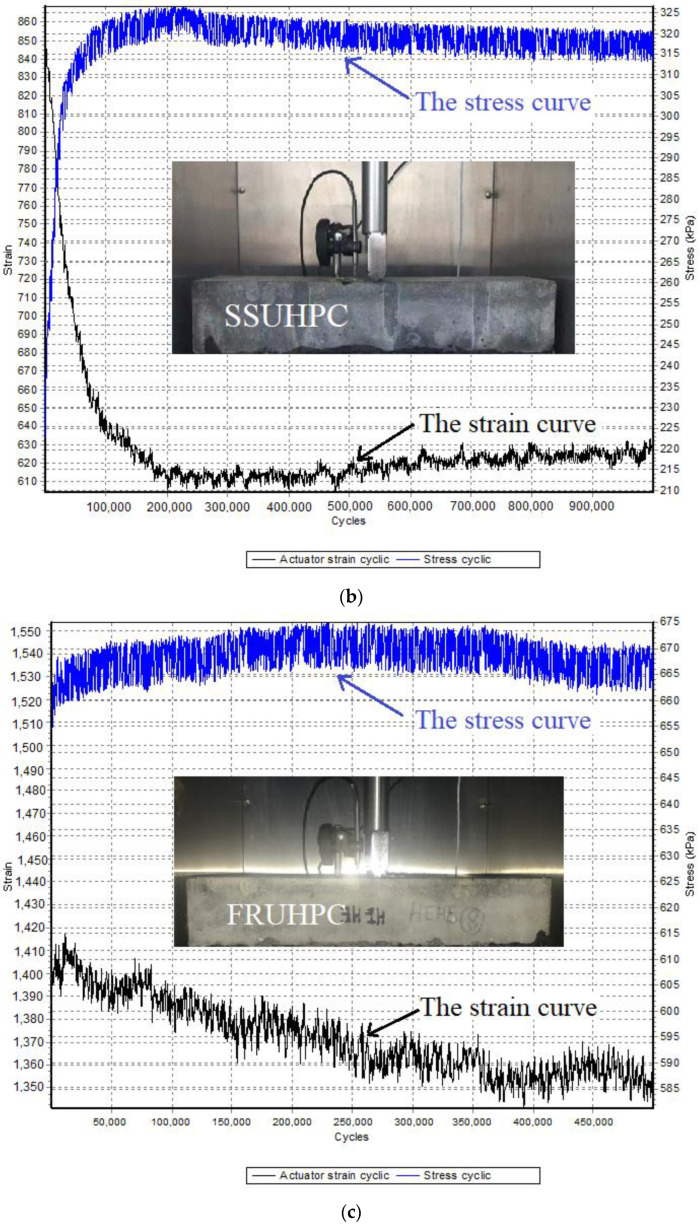

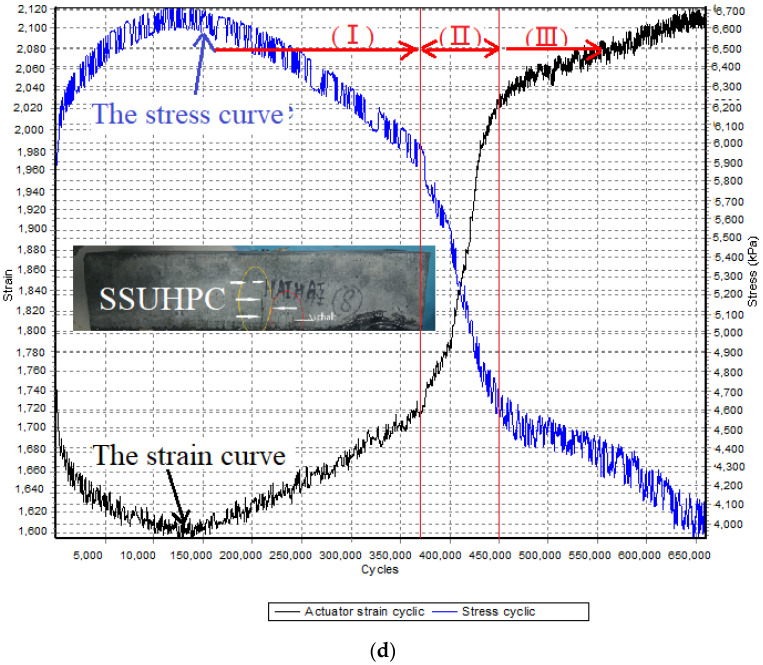
Comparison of stress–strain curves of fatigue tests: (**a**) 1 million loads of FRUHPC when the loading value was 5 kN; (**b**) 1 million loads of SSUHPC when the loading value was 5 kN; (**c**) 500,000 loading times of FRUHPC when the loading value was 10 kN; (**d**) 500,000 loading times of SSUHPC when the loading value was 10 kN.

The fatigue failure process of ultra-high-performance polymer cement mortar presents three stages: crack formation, stable crack development, and crack instability development [54]. Specimen deformation under fatigue load was studied by analyzing the strain–load cyclic relationship curve, as shown in Figure 4, where the black curve is the strain–load cyclic curve, and the blue curve is the stress–load cyclic curve.

When the loading value was 5 kN, the strain of both groups of UHPC remained stable with the increase of loading times, and there was no change during the whole fatigue test, as shown in Figure 4a,b. This demonstrated that both SSUHPC and FRUHPC maintained good fatigue resistance, and no stress failure occured in the 1 million cycle fatigue test. When the loading value was 10 kN, the strain–load cyclic relationship curve remained stable and unchanged for 500,000 loading cycles, and FRUHPC continued to maintain excellent fatigue resistance, as shown in Figure 4c. When the loading value was 10 kN, the strain–load cyclic relationship curve of SSUHPC remained stable during the first 15,000 loading cycles, and the strain tended to change longitudinally during 15,000–35,000 loading cycles. In the process of 35,000–45,000 loading cycles, strain mutation occurred. After 45,000 loading cycles, the strain–load cyclic relationship curve tended to be stable until the end of the test, as shown in Figure 4d. Different from FRUHPC, the fatigue test process of SSUHPC under the loading value of 10 kN presented an obvious three-stage mode of evolution: (I) After a period of stable stress loading, the precursors of stress change start to appear in the structure, this stage was relatively long with unobvious strain; (II) After a long loading cycle time, the strain suddenly increased sharply, and the strain mutation time was short but the change was great; (III) The strain stabilized a short time after mutation until the end of the test.

When the loading value in the fatigue test was 5 kN, no deformation or cracking occurred on the surface of the UHPC materials prepared using freshwater river sand or seawater sea sand, as shown in Figure 4a,b, indicating the good fatigue resistance of both UHPC materials. When the loading value in the fatigue test was 10 kN, there was no damage to FRUHPC after 500,000 times loading, while cracking occurred on the side near the bottom of SSUHPC, as shown in Figure 4d. The fatigue resistance of SSUHPC was inferior to that of FRUHPC.

**Figure 5:**
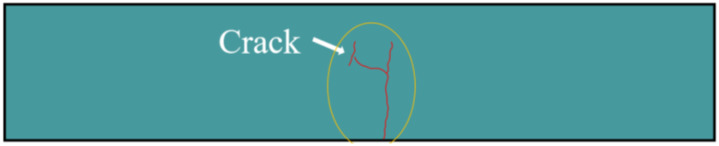
Fatigue failure morphology of SSUHPC: Schematic diagram of fatigue failure.

Figure 5 shows the schematic diagram of fatigue failure in SSUHPC. Before the cracks appeared in the structure, the cement mortar and steel fiber worked together to prevent cracks in the concrete and ensure the structural integrity of SSUHPC. When cracks appeared, the cement mortar in the crack position had lost its stress-bearing role, but the continuous steel fiber structure in SSUHPC was not disconnected and the steel fibers at the cracking place were able to bear the external stress. Therefore, SSUHPC still maintained its structural integrity and demonstrated a certain fatigue resistance when cracking occurred.

#### 3.1.2. Anticorrosion Properties

The carbonization depth of accelerated carbonization in 28 days was 0 and the relative elastic modulus was 100% after 1000 freeze–thaw cycles in fresh water, 3.5 wt% and 7.0 wt% NaCl solutions, as shown in Table 7. The test results showed that both SSUHPC and FRUHPC had excellent carbonation resistance and frost resistance, without any apparent difference. Both SSUHPC and FRUHPC showed good corrosion resistance against chloride ion. The diffusion coefficient of chloride ion in SSUHPC was 1.27 × 10^−12^ m^2^/s and the electric flux was 83 C, slightly lower than FRUHPC. The results of the sulfate erosion test also showed that SSUHPC performed better than FRUHPC.

**Figure 6:**
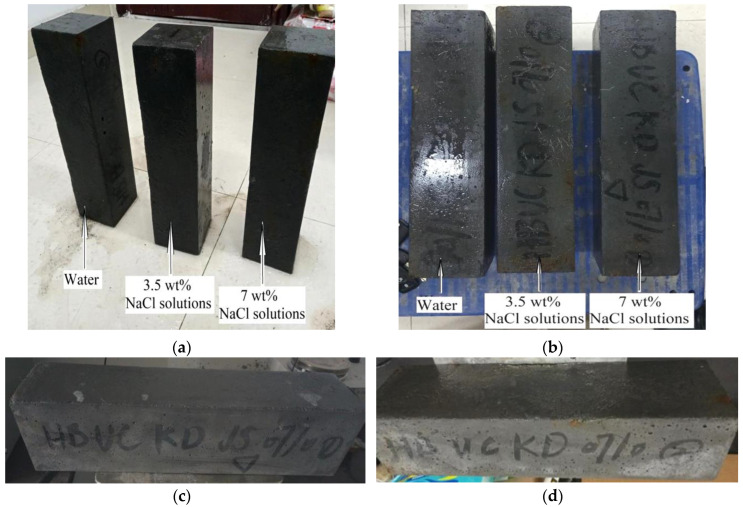
Comparison of SSUHPC/FEUHPC after freezing resistance testing: (**a**) SSUHPC in different solutions after testing; (**b**) FRUHPC in different solutions after testing; (**c**) Appearance of SSUHPC before testing; (**d**) Appearance of SSUHPC after testing in 7 wt% NaCl solutions.

**Figure 7:**
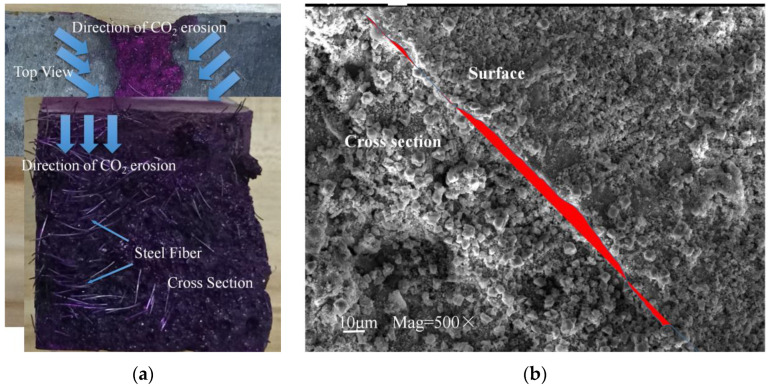

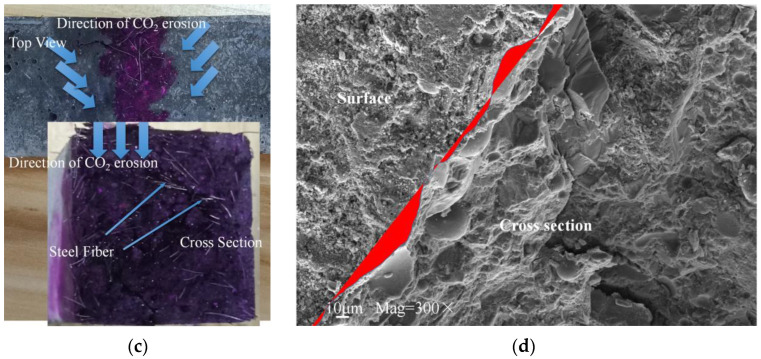
Comparison of SSUHPC and FRUHPC after carbonization: (**a**) Appearance of SSUHPC after carbonization; (**b**) Microstructure of SSUHPC after carbonization; (**c**) Appearance of FRUHPC after carbonization; (**d**) Microstructure of FRUHPC after carbonization.

**Figure 8:**
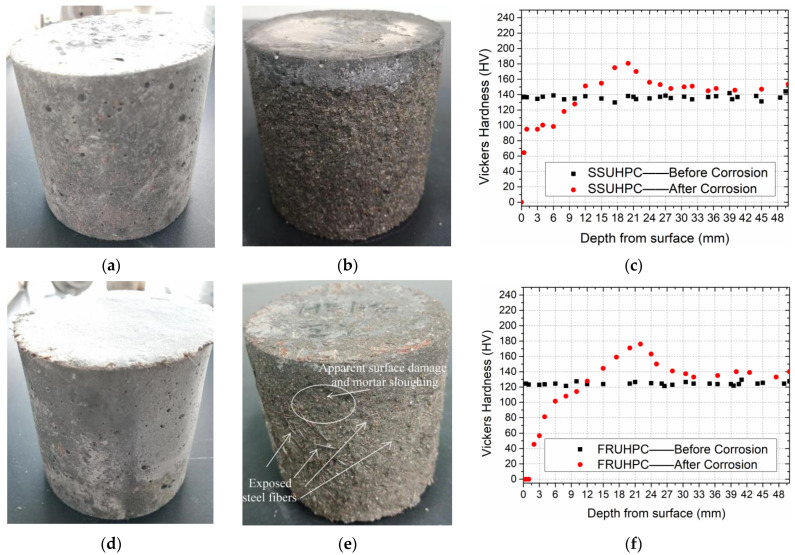
Comparison of SSUHPC and FEUHPC before and after sulfate erosion. (**a**) SSUHPC before sulfate erosion; (**b**) SSUHPC after sulfate erosion; (**c**) Change of Vickers hardness; (**d**) FRUHPC before sulfate erosion; (**e**) FRUHPC after sulfate erosion; (**f**) Change of Vickers hardness.

Figure 6a,b shows the morphology of SSUHPC and FRUHPC after 1000 freeze–thaw cycles. Both SSUHPC and FRUHPC maintained structural integrity without cracks or damage, whether in fresh water, 3.5 wt%, or 7.0 wt% NaCl solution. The appearance changes of SSUHPC before and after frost resistance testing in 7 wt% NaCl solutions are shown in Figure 6c,d. After 1000 freeze–thaw cycles, brown spots appeared on the surface of SSUHPC, which was caused by the iron ions precipitated from steel fibers and generating rust on the surface of the concrete. Figure 7 shows the macroscopic and microscopic morphology of SSUHPC and FRUHPC after carbonization. The carbonation depth of the two kinds of concrete was zero; carbonation was limited to the surface on both kinds of concrete, and carbonation products dominated by CaCO_3_ uniformly covered the surfaces, while no carbonation corrosion was found inside the concrete. The surfaces of both SSUHPC and FRUHPC showed obvious changes under sulfate erosion, as shown in Figure 8. The surface of SSUHPC became rough, without obvious structural defects or leakage of steel fibers after 200 cycles of wetting and drying, as shown in Figure 8b. However, obvious defects appeared and a number of steel fibers leaked out, in addition to the roughness of the surface of FRUHPC, as shown in Figure 8e. The mechanical properties of SSUHPC and FRUHPC after sulfate erosion were investigated by Vickers hardness, as shown in Figure 8c,f. The test results showed that the mechanical properties on the surface of the two samples obviously decreased after sulfate erosion, but the mechanical properties inside were still high.

The sulfate resistance of SSUHPC was obviously better than that of FRUHPC. In conclusion, UHPC prepared with seawater and sea sand had better durability than UHPC prepared with freshwater river sand.

### 3.2. Comparison of Material Characteristics

UHPC is an ultra-high strength cement-based material composed of cement mortar, pores, and steel fibers. The preliminary study found that the mechanics and durability of SSUHPC and FRUHPC were generally at the same level, although there were some differences. Therefore, XRD, SEM, MIP, and X-CT techniques were used to explore the mechanism of the performance differences of the two UHPC materials.

#### 3.2.1. Cement Mortar

**Figure 9:**
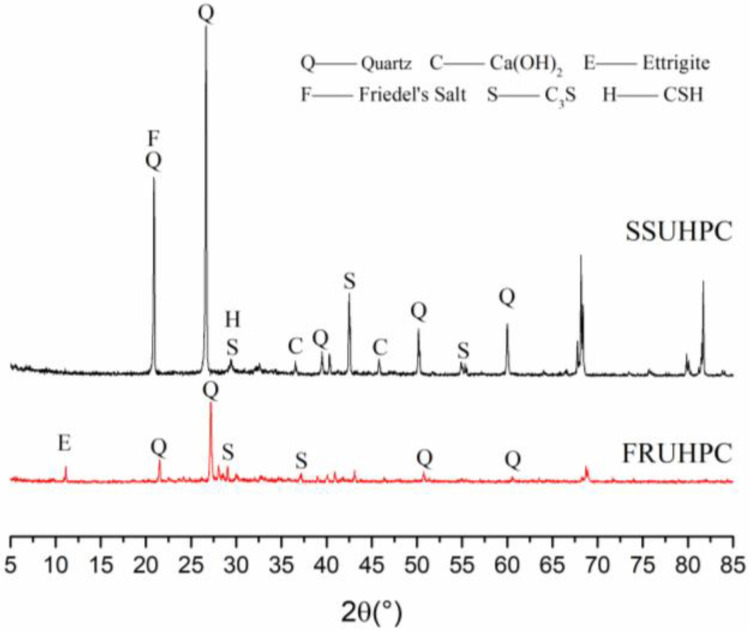
Different groups of XRD maps.

The hydration products of mortar in SSUHPC and FRUHPC were studied by X-ray diffraction test, and the test results are shown in Figure 9. Firstly, there were characteristic diffraction peaks of C_3_S and C-S-H in the XRD patterns, which was due to the incomplete hydration of cement particles, and the coexistence of hydrated and unhydrated particles. Secondly, the characteristic peak of AFt did not appear in SSUHPC. However, AFt appeared in the FRUHPC specimen, which further indicated that the pore structure of cement mortar in FRUHPC was relatively loose. In addition, the presence of chloride ions in seawater and sea sand promoted the dissolution of calcium hydroxide. The hydration of the cement provoked the formation of more mature hydration products, which promoted the hydration of the cement and produced Friedel’s salt.

**Figure 10:**
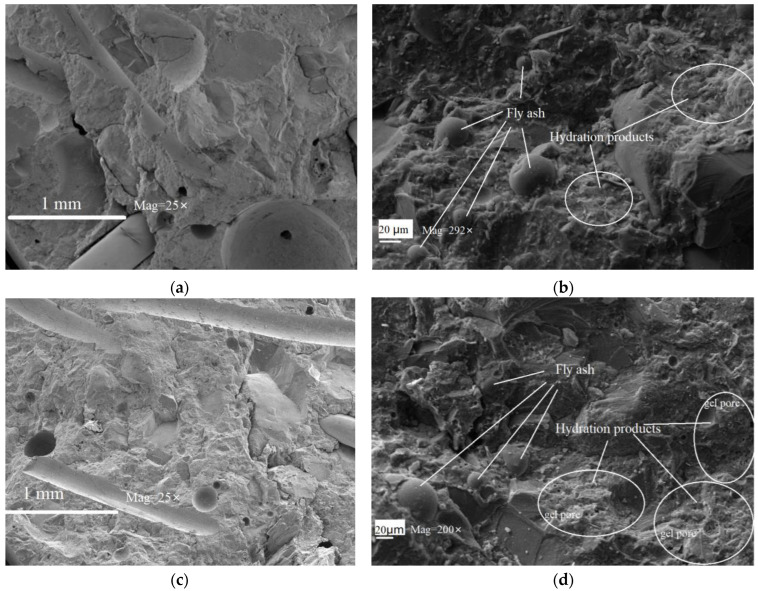
Comparison of SEM microscopic morphology: (**a**) SSUHPC, overall micromorphology; (**b**) SSUHPC, local microscopic morphology characteristics; (**c**) FRUHPC, overall micromorphology; (**d**) FRUHPC, local microscopic morphology characteristics.

The microscopic morphology of SSUHPC and FRUHPC was studied by SEM technology, as shown in Figure 10. The structures of the two concrete materials prepared in this paper were very dense, and the steel fiber was closely combined with the hardened cement mortar, as shown in Figure 10a,c. The fly-ash particles were evenly distributed and the C-S-H gel was distributed on the fly ash particles, which indicated that the fly ash had an obvious pozzolan effect. The hydration products were closely bound to the fly ash, as shown in Figure 10b,d. Although the structures of the two concrete materials were very dense, there were still many pores in the hydration products in FRUHPC, as shown in Figure 10d. Compared with SSUHPC, the mortar in FRUHPC was slightly inferior.

#### 3.2.2. Pore Structure

**Figure 11:**
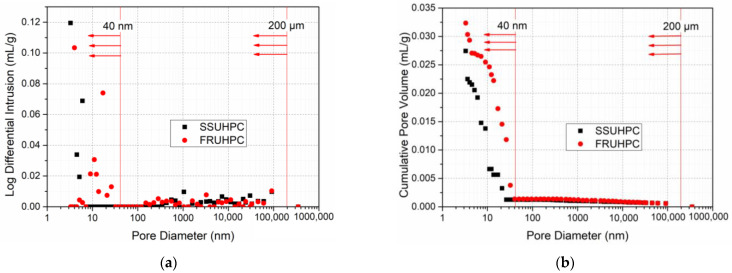
Comparison of pore structure characteristics below 200 μm: (**a**) Integral curve of pore diameter distribution; (**b**) Cumulative pore volume distribution curve.

The pore structure characteristics of mortar in the range of 0~200 μm in SSUHPC and FRUHPC were studied by MIP technology. The results of porosity and cumulative pore volume obtained were shown in Table 8. The porosity of mortar in FRUHPC was low, due to the low W/B ratio, which was 6.9736%. The porosity of SSUHPC was further reduced to 5.9218% after replacing freshwater river sand with seawater sea sand, and the cumulative pore volume was reduced by 15.2%. The pore size distributions of mortar in SSUHPC and FRUHPC are shown in Figure 11a, and the cumulative pore volume distributions are shown in Figure 11b. The pore size distribution of the two types of concrete was mainly concentrated in the range of less than 40 nm; the pore size distribution of FRUHPC was relatively large in the range of 1~40 nm, where the pores were mainly harmless pores (<20 nm) and less harmful pores (20~50 nm); the pore size distribution of SSUHPC was narrower than 20 nm. The low porosity and pore size distribution give SSUHPC and FRUHPC the characteristics of dense pore structure, which was an important factor to ensure the excellent mechanical and durability characteristics of the concrete materials. In conclusion, SSUHPC exhibited better pore structure than FRUHPC in the range of 0~200 μm.

**Figure 12:**
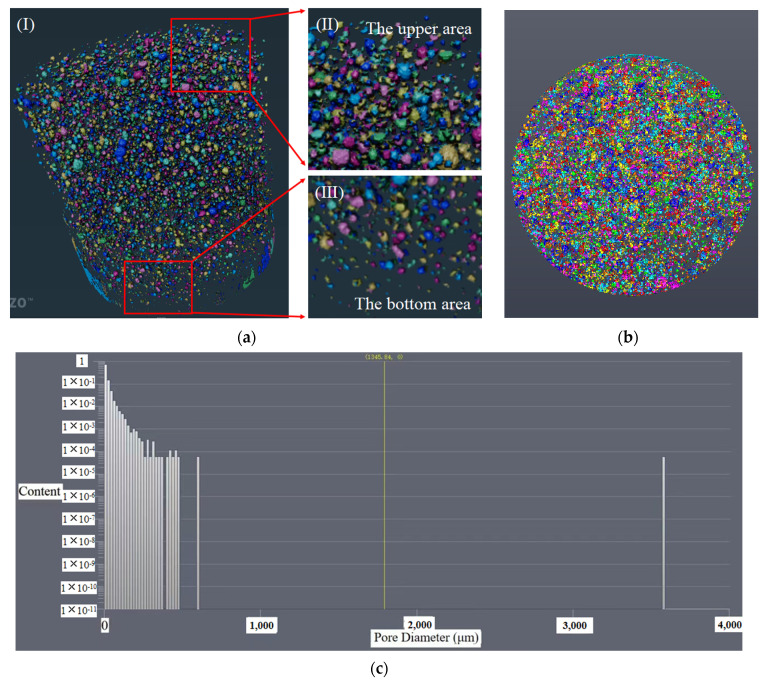
Reconstructed pore structure model of SSUHPC (above 200 μm): (**a**) Analysis of pore structure characteristics. The I was the whole structure, II was the upper area and III was the bottom area; (**b**) Top view of pore structure; (**c**) Pore size distribution of SSUHPC.

**Figure 13:**
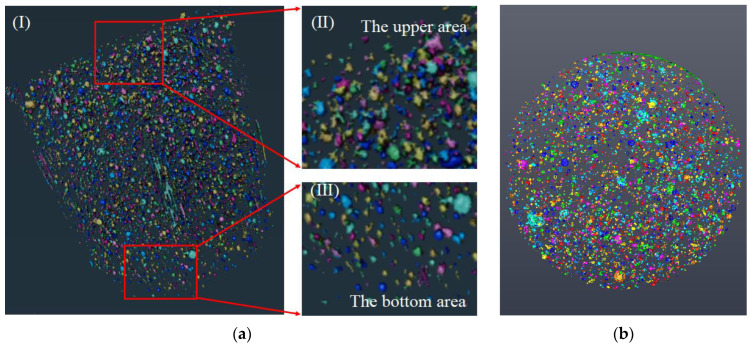

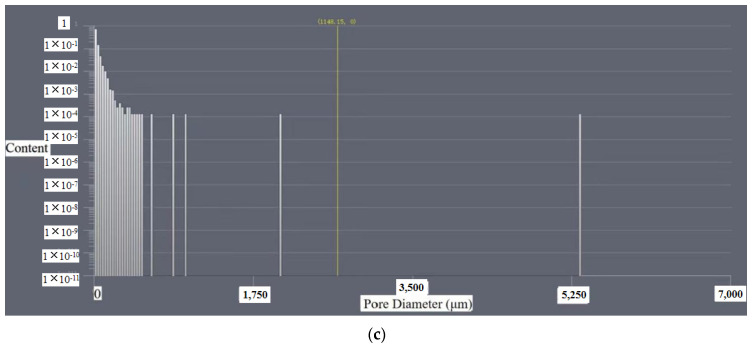
Reconstructed pore structure model of FRUHPC (above 200 μm): (**a**) Analysis of pore structure characteristics. The I was the whole structure, II was the upper area and III was the bottom area; (**b**) Top view of pore structure; (**c**) Pore size distribution of FRUHPC.

Continuous section data of two kinds of concrete were obtained by μX-CT technology, and then the pore structure models of SSUHPC and FRUHPC with pore diameters greater than 200 μm were obtained by Avizo software, as shown in Figure 12 and Figure 13. The porosity of the two types of concrete obtained through modeling calculation is shown in Table 9. Due to the low W/B ratio of UHPC, there were many pores in the concretes made with freshwater river sand and seawater sea sand, as shown in Figure 12a,b and Figure 13a,b. From the distribution characteristics of the pore structure, there were obvious stratifications in the SSUHPC. The size and number of pores in the upper area of the specimen were large (Figure 12(aII)), while the size and number of pores in the bottom area of the specimen were smaller (Figure 12(aIII)). The pores in the middle area of the specimen were relatively uniform, as shown in Figure 12a,b. The pores in FRUHPC were uniformly distributed on the whole, and there was no stratification in its structure, as shown in Figure 13a,b. The results showed that there were 16 905 pores in SSUHPC, and the pore size ranged from 200 to 3 500 μm. FRUHPC had a total of 7 318 pores with an aperture distribution between 200 and 5 300 μm. The porosity of SSUHPC was 1.52%, which was over twice that of FRUHPC (0.60%). FRUHPC exhibited better pore structure than SSUHPC when the pore size was larger than 200 μm.

#### 3.2.3. Fiber Structure

**Figure 14:**
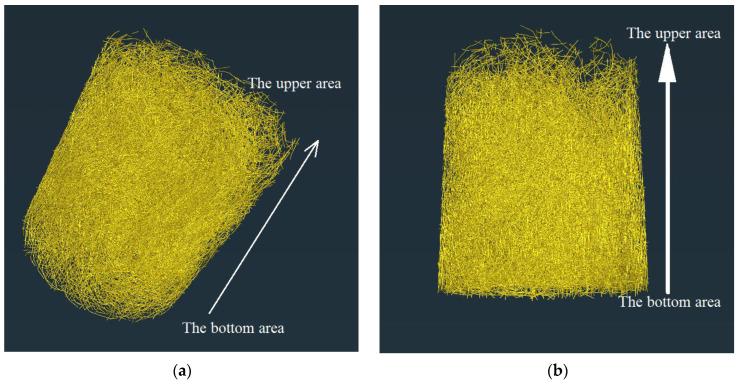

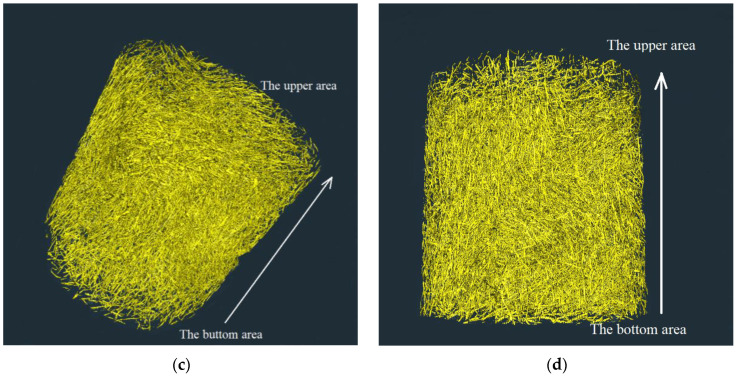
The fiber structures of SSUHPC and FRUHPC: (**a**) SSUHPC, vertical view; (**b**) SSUHPC, side elevation; (**c**) FRUHPC, vertical view; (**d**) FRUHPC, side elevation.

Continuous section data of two kinds of concrete were obtained by μX-CT technology, and fiber structures of SSUHPC and FRUHPC were obtained by Avizo software, as shown in Figure 14. The distribution of steel fibers in SSUHPC and FRUHPC were uniform and dense, and there was no agglomeration phenomenon. From the distribution characteristics of the fibers, precipitation was observed in the SSUHPC prepared with seawater and sea sand. The fibers at the upper areas of the concrete were sparse, and the fibers at the bottom area were compact, as shown in Figure 14a,b. The fibers in FRUHPC were uniformly distributed, and there was no obvious precipitation phenomenon in the SSUHPC structure, as shown in Figure 14c,d. The distribution of steel fiber in FRUHPC was more uniform than that in SSUHPC. The difference of fiber distribution in concrete was also an important factor affecting the mechanical properties of concrete.

## 4. Discussion

**Figure 15:**
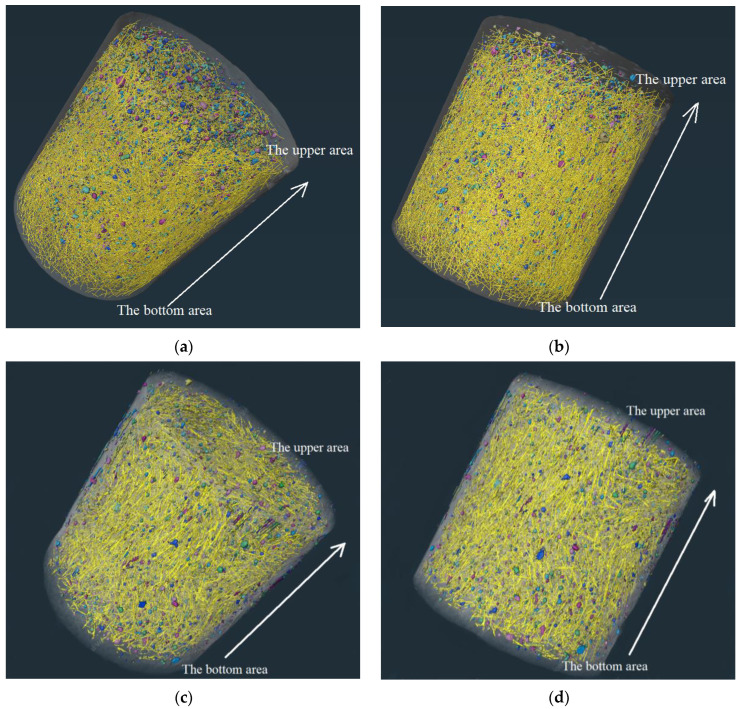
Differences of UHPC prepared with seawater sea sand and freshwater river sand: (**a**) SSUHPC, vertical view; (**b**) SSUHPC, side elevation; (**c**) FRUHPC, vertical view; (**d**) FRUHPC, side elevation.

According to the research above, UHPC with seawater and sea sand demonstrates excellent corrosion resistance, including carbonation resistance, frost resistance, chloride resistance, and sulfate resistance, in which aspects it outperformed FRUHPC. At the same time, we found that the macroscopic mechanical properties of SSUHPC were slightly poor. In order to explore the influence mechanism of material characteristics on mechanical properties and erosion resistance, in this paper, UHPC was separated into mortar, pore, and steel fibers, as shown in Figure 15. Hydration degree of mortar, characteristics of pore structure, and dispersion of steel fibers were important factors affecting the mechanical properties and anticorrosion performance of UHPC materials. The excellent mechanics and anticorrosion performance of UHPC were guaranteed by uniformly dispersed steel fiber, hydrated cement mortar with mature structure, and reasonable air pore structure. Compared with FRUHPC, the mortar in SSUHPC had a higher degree of hydration, and the mortar with a dense and mature hydration structure gave the UHPC material its higher corrosion resistance. Due to the uneven distribution of air pores and steel fibers, the compressive strength and fatigue resistance of SSUHPC were inferior to those of UHPC prepared with freshwater river sand.

For the first time, this paper has verified the possibility of using seawater and sea sand to produce concrete with ultrahigh mechanical properties and anticorrosion performance. SSUHPC was slightly inferior to UHPC prepared from freshwater river sand in terms of its mechanical properties, but it had promising anticorrosion characteristics. In view of these problems, the performance of SSUHPC can be further improved by optimizing the air-pore structure and fiber distribution.

## 5. Conclusions

In this study, seawater and sea sand were used to prepare ultra-high-performance polymer cement mortar. As a comparison, freshwater and river sand were used to prepare FRUHPC. The mechanics and anticorrosion performance of ultra-high-performance polymer cement mortar prepared with seawater and sea sand were comprehensively evaluated, and the mechanism of performance difference between SSUHPC and FRUHPC was revealed. The main conclusions are summarized as follows:

1. The carbonization depth of accelerated carbonization in 28 days was 0 and the relative elastic modulus was 100% after 1000 freeze–thaw cycles in fresh water, 3.5 wt% and 7.0 wt% NaCl solutions. SSUHPC demonstrates excellent corrosion resistance, including carbonation resistance, frost resistance, chloride resistance and sulfate resistance, at all of which it outperformed FRUHPC. Mortar with higher hydration degree and better optimized gel pore characteristics improved the durability SSUHPC compared to FRUHPC. Compared with other seawater sand concrete, SSUHPC has great advantages.
2. The compressive strength of FRUHPC was 173.3 ±8.6 MPa. The compressive strength of SSUHPC prepared with seawater and sea sand reached 162.1 ± 8.1 MPa, which was 93.5% of FRUHPC. Only SSUHPC showed fatigue damage in the fatigue test. However, the Vickers hardness results showed that SSUHPC was slightly harder than FRUHPC. Compared with other seawater sand concrete, SSUHPC has higher value mechanical properties. Although the ultra-high-performance polymer cement mortar prepared with seawater and sea sand revealed excellent mechanical properties, the macroscopic mechanical properties of SSUHPC were lower than those of UHPC with the same mixing ratio of freshwater river sand, due to the disadvantages of air-pore structure and fiber structure.
3. Ultra-high-performance polymer cement materials can be divided into three parts: cement mortar, pores, and steel fibers. A densely structured cement mortar, with continuous and uniform fibers and pore structure promotes the ultra-high mechanical properties and anticorrosion of concrete materials. Although the structures of the two concrete materials were very dense, there were still many pores in the hydration products in FRUHPC. Compared with FRUHPC, the hydration degree of mortar in SSUHPC was higher. The gel pore structure (pore diameter < 200 μm) was more optimized while the air-pore structure (pore diameter ≥ 200 μm) was obviously inferior. The air porosity of SSUHPC was 1.52%, which was over twice that of FRUHPC (0.60%). FRUHPC exhibited better air-pore structure than SSUHPC when the pore size was larger than 200 μm. The fibers in FRUHPC were uniformly distributed on the whole, and there was no obvious precipitation phenomenon apparent in SSUHPC.
4. Compared with UHPC made using freshwater river sand, the preparation of UHPC with seawater and sea sand has its own advantages and disadvantages. In view of the shortcomings of SSUHPC, the performance of UHPC with seawater sand can be further improved by improving the pore structure and fiber structure. In this paper, the performance differences of mechanical and anticorrosion properties in seawater sea-sand and freshwater river-sand ultra-high-performance polymer cement mortars were systematically studied, and the mechanisms of performance difference between the two kinds of concrete materials were revealed. These results lay a solid theoretical foundation for the utilization of sea sand resources and the promotion of seawater sea-sand concrete.

## Figures and Tables

**Table 1:** Basic physical properties of cement.

Setting Time/min		Compressive Strength/MPa		Flexural Strength/MPa		Fineness/%	Density/(g/cm^3^)	Water Consumption for Standard Consistency/%
Initial Setting	Final Setting	3 d	28 d	3 d	28 d			
178	219	23.1	51.2	5.4	9.3	0.8	3.5	29.5

**Table 2:** Basic physical properties of fly ash.

Ratio of Water Demand/%	Fineness/%	Density/(g/cm^3^)	Compressive Strength Ratio/%	Specific Surface Area/(cm^2^/g)	Water Quantity/%	Packing Density/(g/cm^3^)	Normal Consistency/%
94	10	2.1	78	3 400	106	0.78	48

**Table 3:** Basic physical properties of silica fume.

Density/(g/cm^3^)	Specific Surface Area/(cm^2^/g)	Average Particle Size/μm	Bulk Density/(g/mL)
1.6–1.7	(20–28) × 10,000	0.1–0.3	≥0.67

**Table 4:** Basic chemical composition of cement, fly ash and silica fume.

Species	Chemical Composition (wt.%)							
/	CaO	SiO_2_	Al_2_O_3_	MgO	Fe_2_O_3_	Na_2_O	SO_3_	Loss on Ignition
P·O 42.5 cement	61.536	15.404	4.430	0.724	4.906	0.043	2.755	2.243
Silica fume	0.568	97.35	0.337	0.414	0.003	0.101	0.192	2.810
Fly ash	1.5	58	30	2.8	4.3	3.2	0.8	3.310

**Table 5:** Composition of raw materials with different ratios (mass ratio).

Species	Cement	Fly Ash	Silica Fume	Steel Fiber	Polycarboxylic Acid Series of Superplasticizer	Sea Sand	River Sand	Artificial Seawater	Fresh Water
SSUHPC	0.6	0.25	0.15	0.19	0.03	1.4	\	0.14	\
FRUHPC	0.6	0.25	0.15	0.19	0.03	\	1.4	\	0.14

**Table 6:** Specific operation of fatigue test.

Species	Loading Value	Preset Loading Times	Actual Loading Times
FRUHPC	5 kN	1,000,000	1,000,000
FRUHPC	10 kN	1,000,000	500,000
SSUHPC	5 kN	1,000,000	1,000,000
SSUHPC	10 kN	1,000,000	500,000

**Table 7:** Laboratory test results of durability.

Species	Chloride Ion Diffusion Coefficient (RCM Method)	Chloride Ion Permeability (Electricity Method)	Carbonization	Frost Resistance			Resistance of Sulfate Attack
				Fresh Water	3.5 wt% NaCl Solutions	7.0 wt% NaCl Solutions	
**SSUHPC**	1.27 × 10^−12^ m^2^/s	83 C	0 mm	100%	100%	100%	Rough surface, no obvious defect, no steel fiber leakage
**FRUHPC**	1.32 × 10^−12^ m^2^/s	92 C	0 mm	100%	100%	100%	Rough surface, marked defect, steel fiber leakage

**Table 8:** Porosity and cumulative pore volume of SSUHPC and FRUHPC obtained by MIP (below 200 μm).

	SSUHPC	FRUHPC
Porosity (%)	5.9218	6.9736
Cumulative pore volume (mL/g)	0.0274	0.0323

**Table 9:** Porosity of SSUHPC and FRUHPC obtained by μX-CT (above 200 μm).

	SSUHPC	FRUHPC
Porosity (%)	1.52	0.60

## Data Availability

The data underlying this article will be shared on reasonable request from the corresponding author.

## References

[1] Wang S.J., Liu X.Q., Dai Q.F., Li X.J., Chen H. (2003). Distribution characteristics and prospecting direction of Marine sand resources in China. Mar. Geol. Quat. Geol..

[2] Rebentrost M., Cavill B. Reactive powder concrete bridges. Proceedings of the Austroads 6th Bridge Conference: Bridging the Gap.

[3] Blais P.Y., Couture M. (1999). Precast, prestressed pedestrian bridge—World’s first reactive powder concrete structure. PCI J..

[4] Dauriac C. (1997). Special concrete may give steel stiff competition. Seattle Dly. J. Commer..

[5] Dowd W. (1999). Reactive Powder Concrete: Ultra-High Performance Cement Based Composite.

[6] Wang Y.-Z., Wang Y.-B., Zhao Y.-Z., Li G.-Q., Lyu Y.-F. (2020). Experimental study on ultra-high performance concrete under triaxial compression. Constr. Build. Mater..

[7] Alkaysi M., El-Tawil S., Liu Z., Hansen W. (2016). Effects of silica powder and cement type on durability of ultra high performance concrete (UHPC). Cem. Concr. Compos..

[8] Zhu J., Shen D., Xie J., Jin B., Wu S. (2022). Transformation mechanism of carbamic acid elimination and hydrolysis reaction in microbial self-healing concrete. Mol. Simul..

[9] de Larrard F., Sedran T. (1994). Optimization of ultra-high-performance concrete by the use of a packing model. Cem. Concr. Res..

[10] Tayeh B.A., Bakar B.H.A., Johari M.A.M., Voo Y.L. (2012). Mechanical and permeability properties of the interface between normal concrete substrate and ultra high performance fiber concrete overlay. Constr. Build. Mater..

[11] Zhou Z., Qiao P. (2018). Bond behavior of epoxy-coated rebar in ultra-high performance concrete. Constr. Build. Mater..

[12] Hor Y., Teo W., Kazutaka S. (2017). Experimental investigation on the behaviour of reinforced concrete slabs strengthened with ultra-high performance concrete. Constr. Build. Mater..

[13] Naeimi N., Moustafa M.A. (2021). Compressive behavior and stress-strain relationships of confined and unconfined UHPC. Constr. Build. Mater..

[14] Yang J., Peng G.-F., Zhao J., Shui G.-S. (2019). On the explosive spalling behavior of ultra-high performance concrete with and without coarse aggregate exposed to high temperature. Constr. Build. Mater..

[15] Yu R., Zhou F., Yin T., Wang Z., Ding M., Liu Z., Leng Y., Gao X., Shui Z. (2020). Uncovering the approach to develop ultra-high performance concrete (UHPC) with dense meso-structure based on rheological point of view: Experiments and modeling. Constr. Build. Mater..

[16] Dagenais M.-A., Massicotte B., Boucher-Proulx G. (2018). Seismic Retrofitting of Rectangular Bridge Piers with Deficient Lap Splices Using Ultrahigh-Performance Fiber-Reinforced Concrete. J. Bridge Eng..

[17] Semendary A.A., Walsh K.K., Steinberg E.P. (2017). Early-Age Behavior of an Adjacent Prestressed Concrete Box-Beam Bridge Containing UHPC Shear Keys with Transverse Dowels. J. Bridge Eng..

[18] Zhou M., Lu W., Song J., Lee G.C. (2018). Application of Ultra-High Performance Concrete in bridge engineering. Constr. Build. Mater..

[19] Norhasri MS M., Hamidah M.S., Fadzil A.M., Megawati O. (2016). Inclusion of nano metakaolin as additive in ultra high performance concrete (UHPC). Constr. Build. Mater..

[20] Tanarslan H.M. (2017). Flexural strengthening of RC beams with prefabricated ultra high performance fibre reinforced concrete laminates. Eng. Struct..

[21] Cho D.-O. (2005). Challenges to sustainable development of marine sand in Korea. Ocean Coast. Manag..

[22] Kim T.-G. (2009). Efficient management of marine resources in conflict: An empirical study of marine sand mining, Korea. J. Environ. Manag..

[23] Shi M.P., Lu F.H. (2004). Study on the application of desalinated sea sand in high performance concrete. Concrete.

[24] Amey S.L., Johnson D.A., Miltenberger M.A., Farzam H. (1998). Predicting the Service Life of Concrete Marine Structures: An Environmental Methodology. Struct. J..

[25] Liu T.-K., Sheu H.-Y., Tseng C.-N. (2013). Environmental impact assessment of seawater desalination plant under the framework of integrated coastal management. Desalination.

[26] Zhu J., Shen D., Jin B., Wu S. (2022). Theoretical investigation on the formation mechanism of carbonate ion in mi-crobial self-healing concrete: Combined QC calculation and MD simulation. Constr. Build. Mater..

[27] Li Q., Geng H., Huang Y., Shui Z. (2015). Chloride resistance of concrete with metakaolin addition and seawater mixing: A comparative study. Constr. Build. Mater..

[28] Guo M., Hu B., Xing F., Zhou X., Sun M., Sui L., Zhou Y. (2020). Characterization of the mechanical properties of eco-friendly concrete made with untreated sea sand and seawater based on statistical analysis. Constr. Build. Mater..

[29] Li Y.T., Zhou L., Jiang M., Shao L. (2013). Experimental Study on Mechanical Property of Concrete Based on Seawater and Sea Sand. Adv. Mater. Res..

[30] Limeira J., Etxeberria M., Agulló L., Molina D. (2011). Mechanical and durability properties of concrete made with dredged marine sand. Constr. Build. Mater..

[31] Liu W., Xie Y.J., Dong B.Q., Xing F. (2014). Study on the properties of sea sand and mechanical properties of sea sand concrete. Bull. Chin. Ceram. Soc..

[32] He X., Zhou J. (2020). Mechanical characteristics of sea-sand concrete in simulated marine environment. Constr. Build. Mater..

[33] Liu J., Fan X., Liu J., Jin H., Zhu J., Liu W. (2021). Investigation on mechanical and micro properties of concrete incorporating seawater and sea sand in carbonized environment. Constr. Build. Mater..

[34] Shi Z., Shui Z., Li Q., Geng H. (2015). Combined effect of metakaolin and sea water on performance and microstructures of concrete. Constr. Build. Mater..

[35] Xiao J., Qiang C., Nanni A., Zhang K. (2017). Use of sea-sand and seawater in concrete construction: Current status and future opportunities. Constr. Build. Mater..

[36] Zhang Q., Xiao J., Zhang P., Zhang K. (2019). Mechanical behaviour of seawater sea-sand recycled coarse aggregate concrete columns under axial compressive loading. Constr. Build. Mater..

[37] Dasar A., Patah D., Hamada H., Sagawa Y., Yamamoto D. (2020). Applicability of seawater as a mixing and curing agent in 4-year-old concrete. Constr. Build. Mater..

[38] Li T.Y., Liu X.Y., Zhang Y.M. (2020). Carbonization Mechanism of Reactive Powder Concrete with Sea-water and Sea Sand. Mater. Rep..

[39] Li T.Y., Zhang Y.M., Liu X.Y. (2019). Research on the preparation and durability of brine marine sand high performence concrete. Concrete.

[40] Zhang K., Li T.Y., Liu X.Y., Yang S.C., Wang D.Z., Li W.H. (2020). Research on High Temperature Resistance of Seawater Sand High Performance Concrete. Fly Ash Compr. Util..

[41] Li T., Liu X., Zhang Y., Yang H., Zhi Z., Liu L., Ma W., Shah S.P., Li W. (2020). Preparation of sea water sea sand high performance concrete (SHPC) and serving performance study in marine environment. Constr. Build. Mater..

[42] Chotard T.J., Boncoeur-Martel M.P., Smith A., Dupuy J.P., Gault C. (2003). Application of X-ray computed tomography to characterise the early hydration of calcium aluminate cement. Cem. Concr. Compos..

[43] Gallucci E., Scrivener K., Groso A., Stampanoni M., Margaritondo G. (2007). 3D experimental investigation of the microstructure of cement pastes using synchrotron X-ray microtomography (μCT). Cem. Concr. Res..

[44] Promentilla M.A.B., Sugiyama T., Hitomi T., Takeda N. (2009). Quantification of tortuosity in hardened cement pastes using synchrotron-based X-ray computed microtomography. Cem. Concr. Res..

[45] Sugiyama T., Promentilla M.A.B., Hitomi T., Takeda N. (2010). Application of synchrotron microtomography for pore structure characterization of deteriorated cementitious materials due to leaching. Cem. Concr. Res..

[46] Zhu J., Shen D., Xie J., Tang C., Jin B., Wu S. (2021). Mechanism of urea decomposition catalyzed by *Sporosarcina pasteurii* urease based on quantum chemical calculations. Mol. Simul..

[47] Wang R., Gao X., Zhang J., Han G. (2018). Spatial distribution of steel fibers and air bubbles in UHPC cylinder determined by X-ray CT method. Constr. Build. Mater..

[48] Zhu J., Shen D., Wu W., Jin B., Wu S. (2021). Hydration inhibition mechanism of gypsum on tricalcium aluminate from ReaxFF molecular dynamics simulation and quantum chemical calculation. Mol. Simul..

[49] Shi F., Li T., Wang W., Liu R., Liu X., Tian H., Liu N. (2021). Research on the Effect of Desert Sand on Pore Structure of Fiber Reinforced Mortar Based on X-CT Technology. Materials.

[50] Jiang J., Yuan Y. (2012). Prediction model for the time-varying corrosion rate of rebar based on micro-environment in concrete. Constr. Build. Mater..

[51] Zhou J., He X., Zhang L. (2020). CT characteristic analysis of sea-sand concrete exposed in simulated marine environment. Constr. Build. Mater..

[52] Sun X., Li T., Shi F., Liu X., Zong Y., Hou B., Tian H. (2022). Sulphate Corrosion Mechanism of Ultra-High-Performance Concrete (UHPC) Prepared with Seawater and Sea Sand. Polymers.

[53] Wang D., Shi C., Wu Z., Xiao J., Huang Z., Fang Z. (2015). A review on ultra high performance concrete: Part II. Hydration, microstructure and properties. Constr. Build. Mater..

[54] Birol Fitik R.N., Konrad Z. Fatigue Behaviour of Utral High-Performance Concrete under Cylic Strsee Reversal Loading. Proceedings of the Second International Symposium of UHPC.

